# State of the Art of Innate Immunity—An Overview

**DOI:** 10.3390/cells11172705

**Published:** 2022-08-30

**Authors:** Silvia Fischer, Elisabeth Deindl

**Affiliations:** 1 Institute of Biochemistry, Justus-Liebig-University, 35392 Giessen, Germany; 2 Walter-Brendel-Centre of Experimental Medicine, University Hospital, Ludwig-Maximilians-University, 81377 Munich, Germany; 3 Biomedical Center, Institute of Cardiovascular Physiology and Pathophysiology, Ludwig-Maximilians-University, Planegg-Martinsried, 82152 Munich, Germany

The innate immune system is the first line of defense against bacterial and viral infections and sterile inflammation through the recognition of pathogen-associated molecular patterns (PAMPs) as well as danger-associated molecular patterns (DAMPs) by pathogen-recognition receptors (PRRs), and produces proinflammatory and antiviral cytokines and chemokines [1]. 

This Special Issue of *Cells* is devoted to many aspects of innate immunity and gives an overview of different DAMPs, immune cells, special mechanisms, and therapeutic options for treating diseases related to chronic inflammation or infections.

One of the well-known DAMPs is the high-mobility group box 1 protein (HMGB1), which is either passively released by dying cells or actively secreted by immune and other cells and was described as implicated in both stimulating and inhibiting innate immunity. Andersson et al. reported that the pro- and anti-inflammatory activities of HMGB1 depend on post-translational modification of its disulfide bonds by binding to different extracellular cell surface receptors either directly or as a cofactor of PAMPs [2]. 

Another DAMP, extracellular ribosomal RNA, which is released under pathological conditions from damaged tissue, acts synergistically with Toll-like receptor 2 ligands, inducing the release of cytokines in a nuclear factor kappa B-dependent manner in vitro as well as in vivo. Grote et al. suggest that extracellular RNA might sensitize Toll-like receptor 2 to enhance the immune response under pathological conditions and therefore might serve as a new target for the treatment of bacterial or viral infections [3].

Arnholdt et al. demonstrate that cells related to innate immunity and influencing immunoregulatory and inflammatory processes, such as gamma delta T cells, play an important role in angiogenesis and tissue generation. By using a femoral artery ligation model in mice, depletion of this subset of T cells was demonstrated to impair angiogenesis, increase the number of leukocytes and inflammatory M1-like macrophages, and promote the formation of neutrophil extracellular traps (NETs) [4]. 

The topic of autoinflammation is also covered in this Special Issue. The review of P. Georgel provides some examples of autoimmune/autoinflammatory diseases caused by the deregulated expression of type I interferons and interleukin-1β. The role of interleukin-1 and type I interferons and their crosstalk in autoinflammatory diseases such as rheumatic diseases are analyzed to reveal novel therapeutic opportunities [5].

Gullet et al. discuss the key components of programmed cell death pathways and highlight the plasticity of pyroptosis, apoptosis, and necroptosis as well as significant crosstalk among these pathways. The concept of PANoptosis, an inflammatory cell death pathway that integrates components of different cell death pathways and is implicated in driving innate immune responses and inflammation, is explained [6]. 

A review by Papendorf et al. provides a comprehensive overview of molecular pathogenesis disorders caused by proteostasis perturbations, and current knowledge of the various mechanisms by which impaired proteostasis promotes autoinflammation is summarized [7].

To investigate the crosstalk between coagulation and innate immunity, the effect of thrombin on macrophage polarization is investigated by Ukan et al. Results demonstrate that thrombin induces an anti-inflammatory phenotype in macrophages, which shows similarities to as well as differences from the classical M2 polarization states regarding the expression of secreted modular Ca^2+^-binding protein [8].

To investigate insect innate immunity, the in vitro cultivation of primary hemocytes from *D. Suzuki* third-instar larvae is described by Carrau et al. as a valuable tool for investigating hemocyte-derived effector mechanisms against pathogens, particularly for the formation of extracellular traps [9]. 

Drugs such as ganciclovir and its pro-drug valganciclovir are often used to treat viremic patients transfected with, e.g., human cytomegalovirus (HCMV). Results from Landázuri now suggest that binding and signaling through endothelin receptor B (ETBR) is crucial for viral replication and that selected ETBR blockers inhibit HCMV infections [10].

Lin et al. report that albumin attenuates chronic liver diseases (CLDs) via alleviating inflammation of Kupffer cells caused by bacterial products, which might provide a compelling rationale for albumin therapy in patients with CLDs [11].
